# Supplementary material for: Size‐Modulated Mesoderm‐Endoderm Divergence and Myocardial Cavitation in Micropatterned Cardioids
Source: Adv Sci (Weinh). 2026 Mar 16;13(30):e15661. doi: 10.1002/advs.202515661 (PMC13248853; doi:10.1002/advs.202515661)
Supplement: Supplementary file 1 — Supporting File 1: advs74868‐sup‐0001‐SuppMat.pdf [file ADVS-13-e15661-s002.pdf]

SUPPLEMENTARY INFORMATION

Size-Modulated Mesoderm-Endoderm Divergence and Myocardial Cavitation in Micropatterned Cardioids

Plansky Hoang[1,2]*, David W. McKellar[3]*, Andrew Kowalczewski[1,2]*, Nhu Y. Mai[1,2], Meng Chai[1,2], Xiaojun L. Lian[4,5,6], Yi Zheng[1,2], Jeffrey Amack[7], Nathan Tucker[8], Iwijn De Vlaminck[3], Huaxiao Yang[9], Benjamin D. Cosgrove[3#], Zhen Ma[1,2#]

[1]Department of Biomedical and Chemical Engineering, Syracuse University, Syracuse, NY, USA.
[2]BioInspired Syracuse Institute for Material and Living Systems, Syracuse, NY, USA.
[3]Meinig School of Biomedical Engineering, Cornell University, Ithaca, NY, USA.
[4]Department of Biomedical Engineering, Pennsylvania State University, University Park, PA, USA
[5]Department of Biology, Pennsylvania State University, University Park, PA, USA
[6]The Huck Institutes of the Life Sciences, Pennsylvania State University, University Park, PA, USA
[7]Department of Cell and Developmental Biology, Upstate Medical University, Syracuse, NY, USA
[8]Department of Pharmacology, Upstate Medical University, Syracuse, NY, USA
[9]Department of Biomedical Engineering, University of North Texas, Denton, TX

*These authors contributed equally.

[#]Corresponding Authors:
Benjamin D. Cosgrove <bdc68@cornell.edu>
Zhen Ma <zma112@syr.edu>

Confocal Microscopy

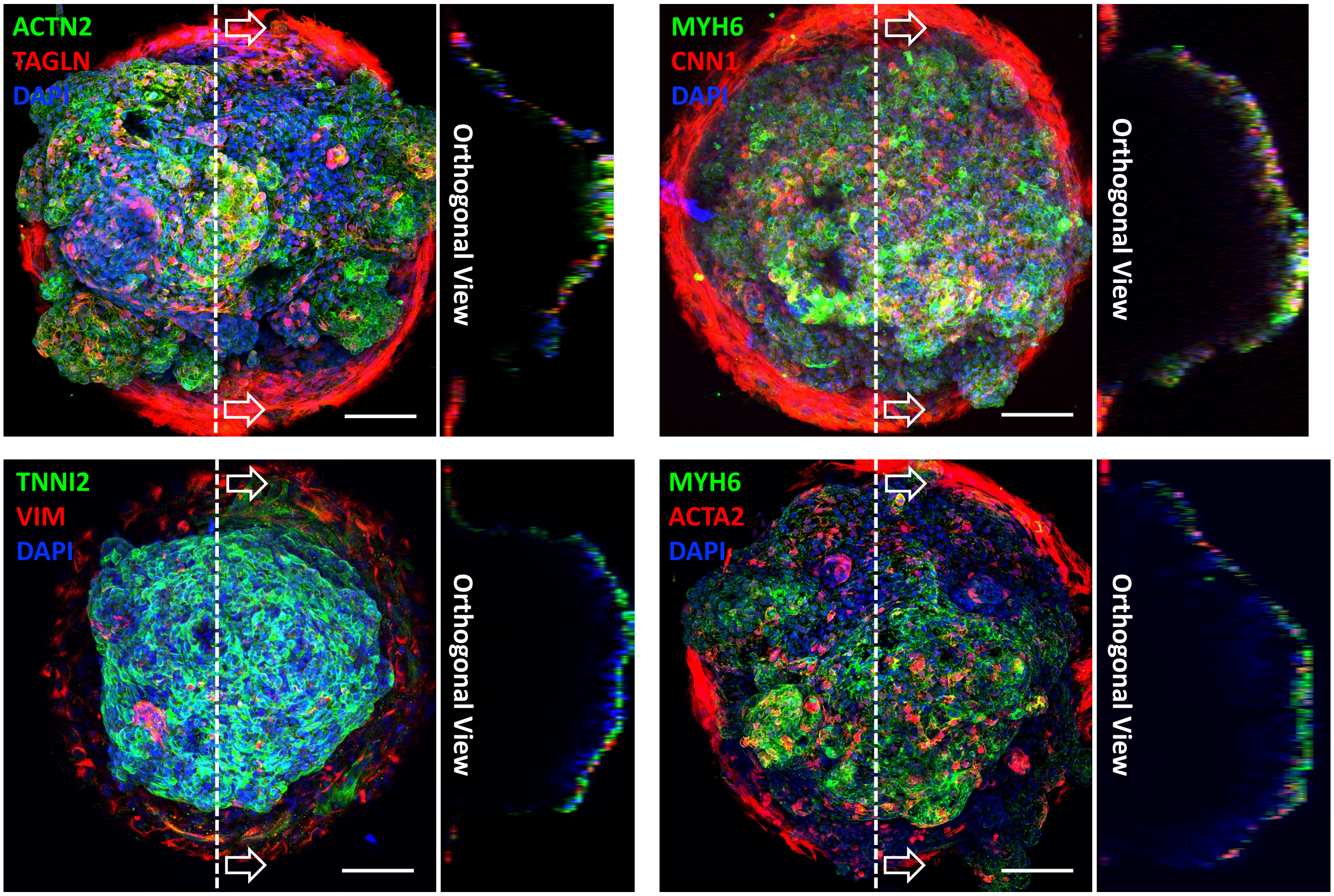

Figure S1. Representative 3D reconstruction images of confocal microscopy of micropatterned cardioids (related to Figure 1). The micropatterned cardioids were derived from WTC hiPSC line without any fluorescent reporter, whole mounted without cryo-sectioning, and stained with cardiomyocyte markers (TNNI2, ACTN2, and MYH6) and stromal cell markers (TAGLN, CNN1, VIM, and ACTA2). Scale: 100 μm.

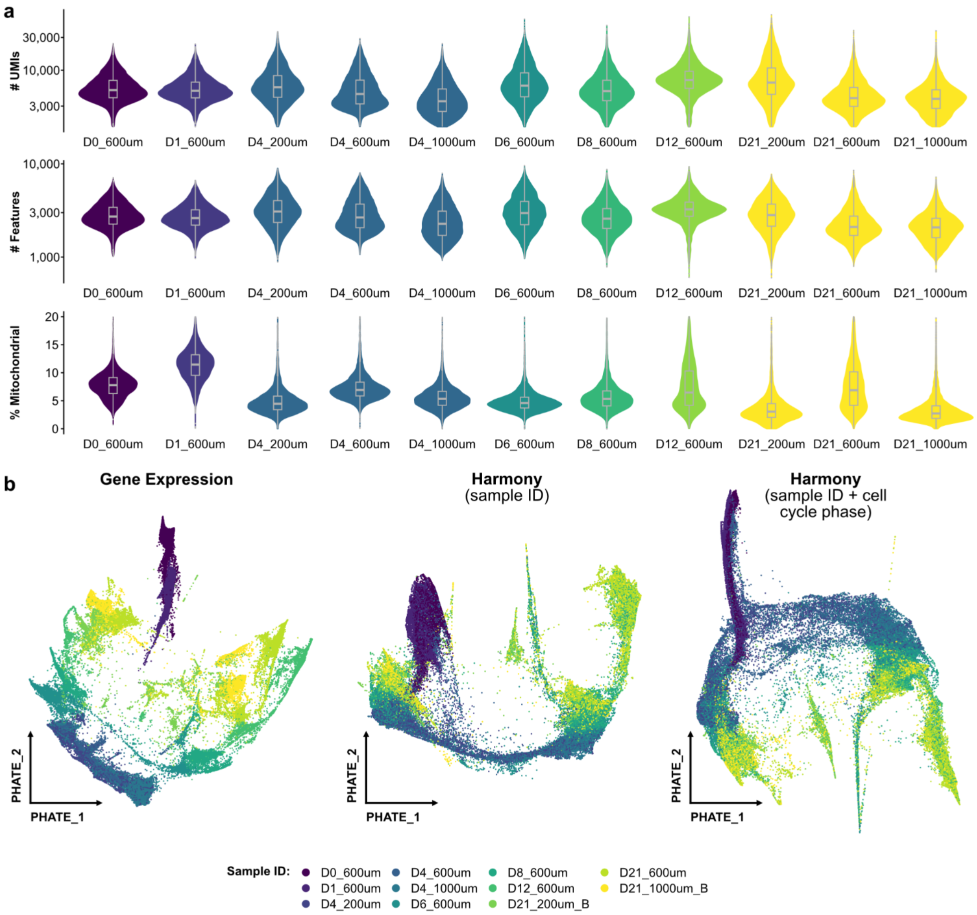

Figure S2. Quality control of scRNA-seq dataset (Related to Figure 2). (a) The number of unique molecular identifier (UMI), number of features, and % of mitochondrial genes were analyzed as quality control metrics in preprocessing steps. (b) Comparison of trajectory construction with no batch correction, batch correction based on sample ID, and batch correction based on sample ID and cell cycle phases.

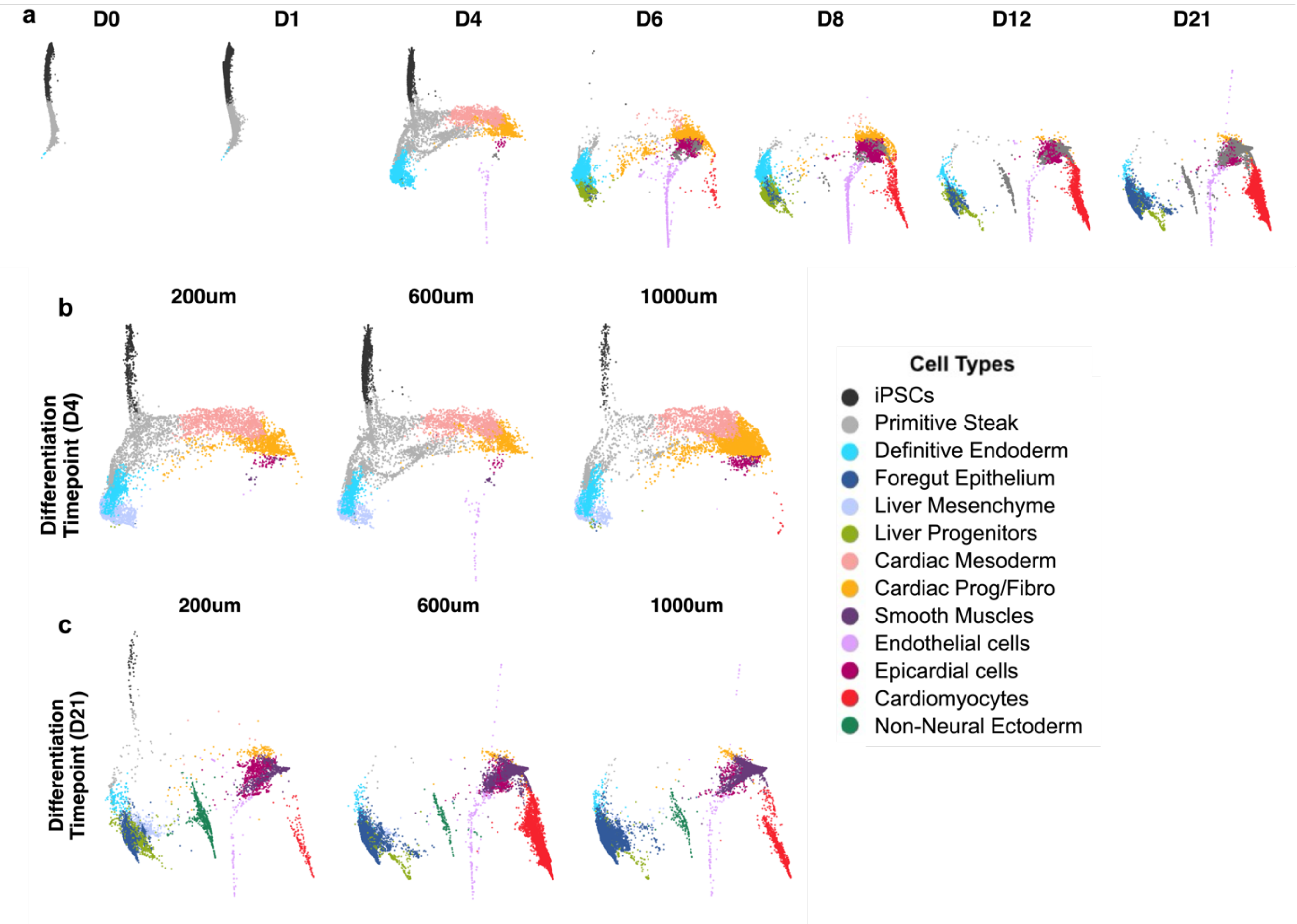

Figure S3. PHATE Embedding for Individual Samples (Related to Figure 2). (a) PHATE embedding depicting developmental progression at seven time points during 600 µm cardioid differentiation. (b) PHATE embedding at Day 4, illustrating mesoderm-endoderm divergence from three different pattern sizes. (c) PHATE embedding at Day 21, visualizing heart-foregut co-development in cardioids derived from three different pattern sizes.

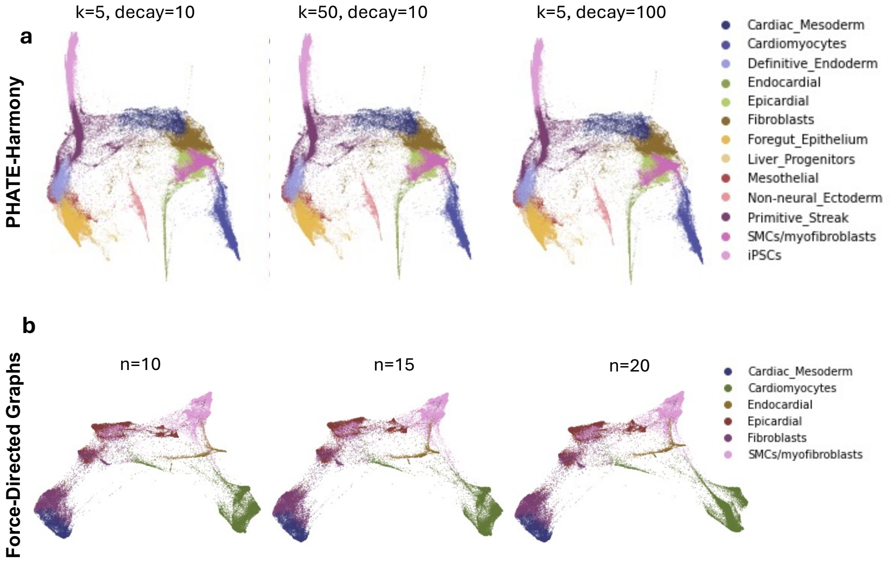

Figure S4. Parameterization for PHAT and PAGA/force-directed graph (Related to Figure 2 and 3). (a) PHATE embeddings were optimized based on nearest neighbors (k) and diffusion decays (decay). (b) PAGA analysis was optimized based on the numbers of neighbors (n). Across the parameter space, the global manifold structure and relative branching relationships were preserved, with only minor local variations in the resulting embedding geometry.

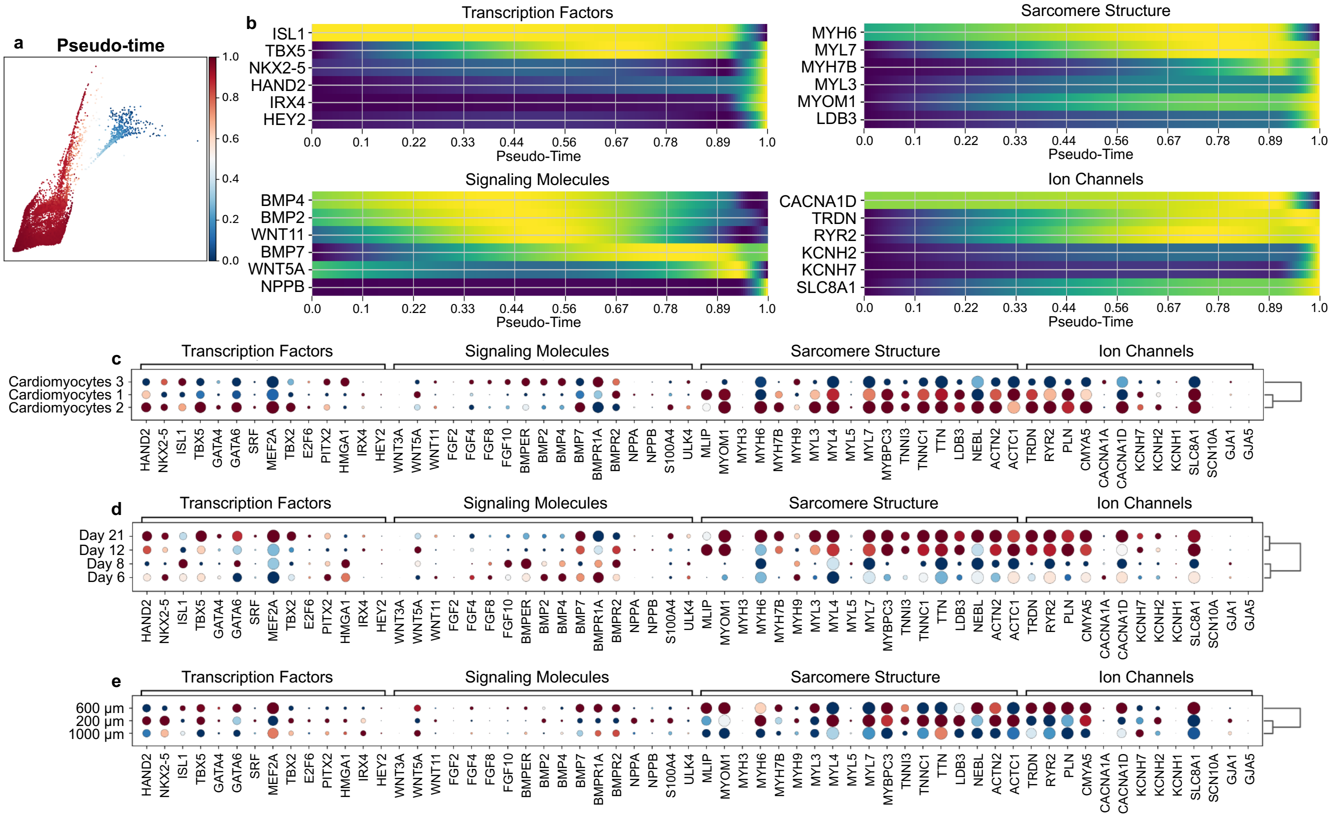

Figure S5. Profile cardiomyocyte differentiation progress (Related to Figure 3). (a) Pseudo-time was constructed for cardiomyocyte clusters using CellRank. (b) Pseudo-time progression of key cardiomyocyte genes for transcription factors, signaling molecules, sarcomere structures, and ion channels. Gene expression comparison for (c) three cardiomyocyte clusters from PAGA-guided force-directed dimensions, (d) four differentiation time points during cardioid differentiation, and (e) Day 21 cardioids generated from three different pattern sizes.

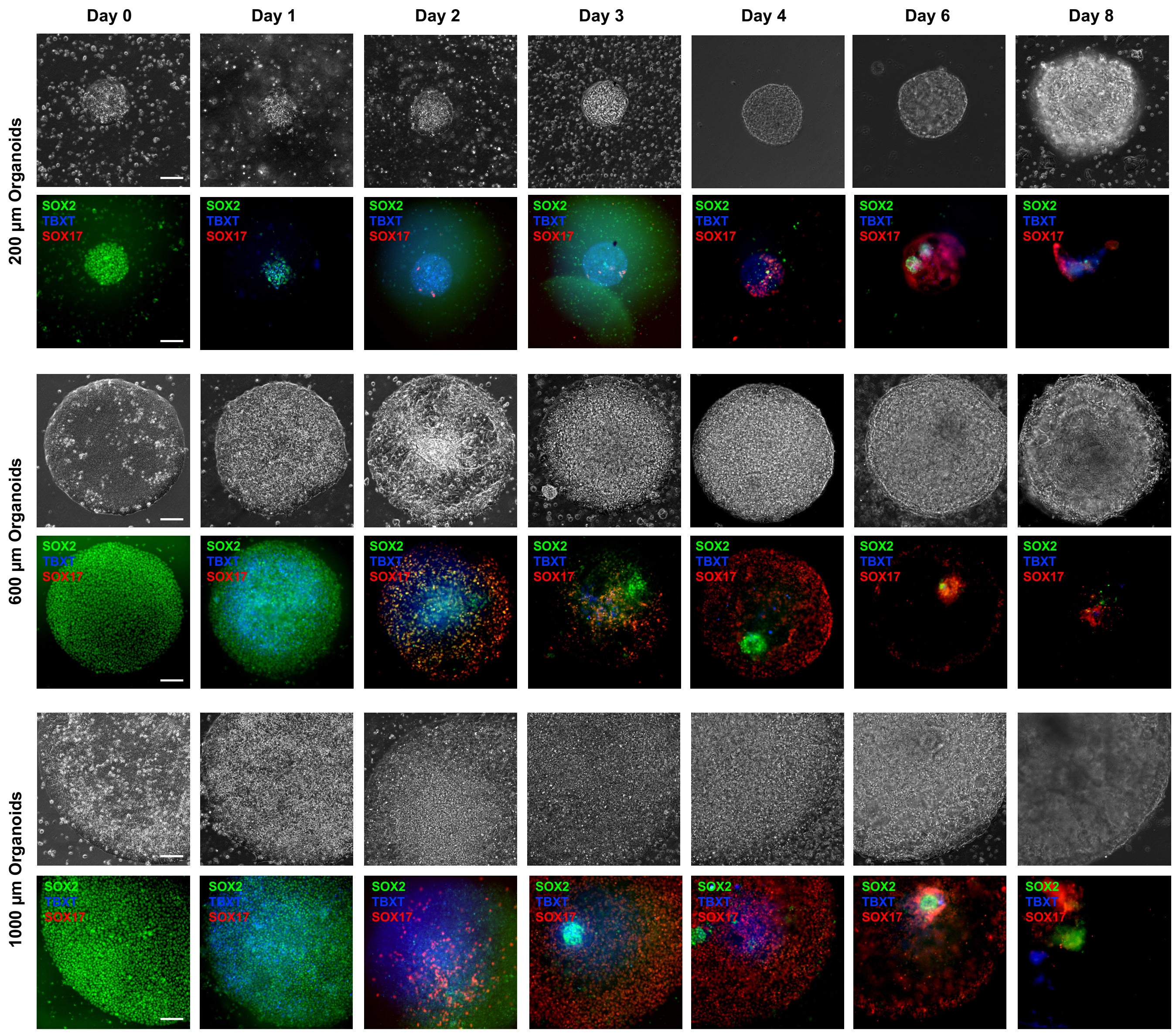

Figure S6. Cell fate transition during mesoderm-endoderm divergence (related to Figure 4). Representative images of brightfield morphology and fluorescence signals for SOX2–mCitrine, BRA–mCerulean, and SOX17–tdTomato in micropatterned RUES2-GLR hESCs with different sizes across different time points (Day 0–Day 8). The images illustrate the decline of SOX2 expression, with TBXT (BRA) and SOX17 emerging at Day 1 and peaking at Day 2.

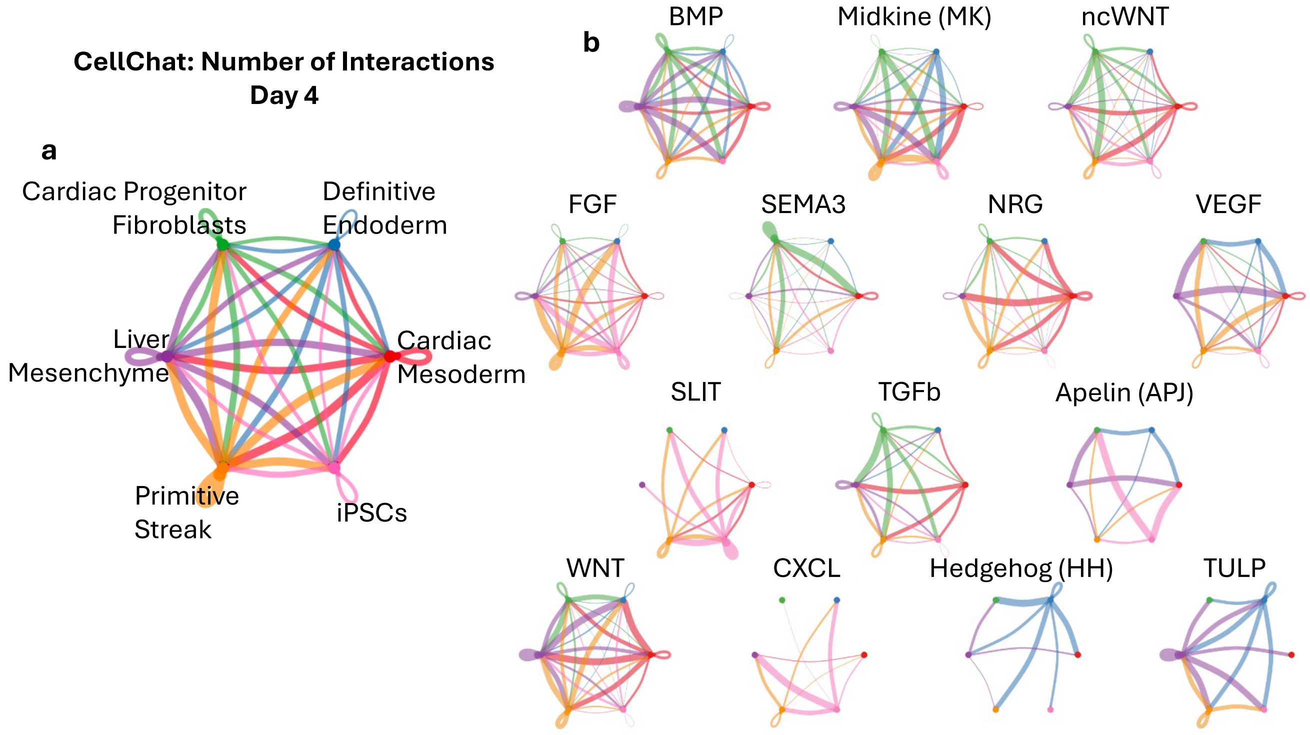

Figure S7. Ligand-receptor analysis on Day 4 during mesoderm-endoderm divergence (related to Figure 4). (a) Cell-cell interaction networks for all detected signaling pathways. (b) Cell-cell interactions highlighting selected embryogenesis-related signaling pathways.

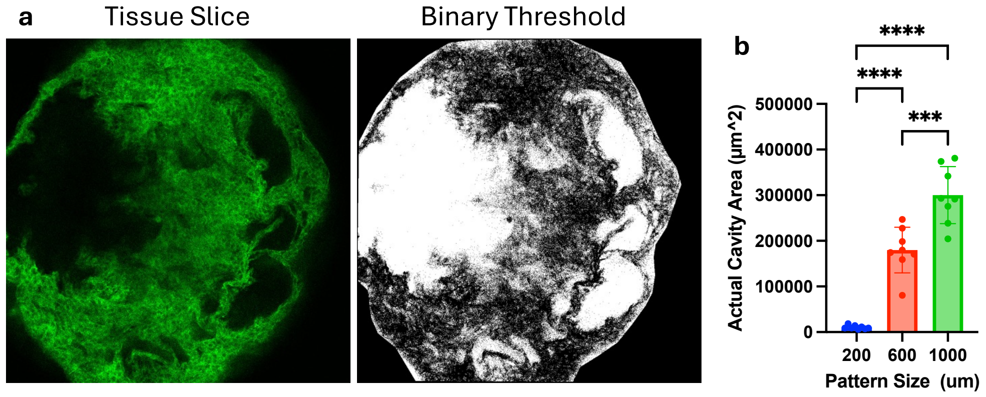

Figure S8. Quantification of cardioid cavitation (Related to Figure 5). (a) Representative images and binary processing used to estimate cavity area from a single optical slice of a cardioid. (b) Cardioid size varied substantially across the three pattern sizes, resulting in large differences in absolute cavity area. Quantitative analysis revealed a significant increase in cavity area with increasing pattern size. Sample size > 6 cardioids from three independent differentiation. Statistical analysis: one-way ANOVA with post-hoc Tukey analysis (*p < 0.05, **p<0.01, ***p<0.001, ****p<0.001).

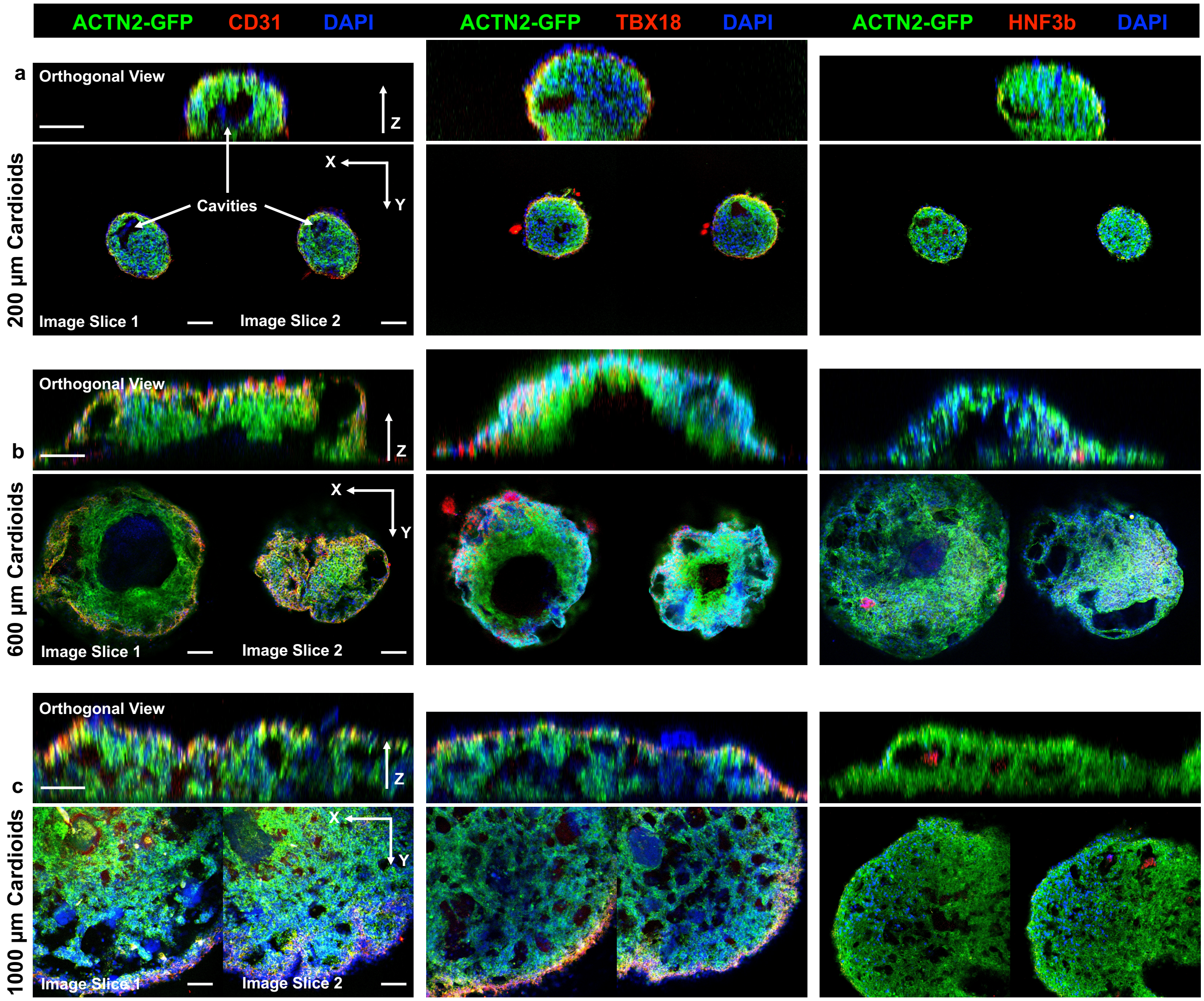

Figure S9. Cavitation inside Day 21 cardioids generated from different pattern sizes (related to Figure 5). Representative two-photon microscopy images of micropatterned cardioids generated from ACTN2-GFP reporter hiPSC line and stained with endothelial marker (CD31), epicardial marker (TBX18), and foregut marker (HNF3b). 3D reconstructed orthogonal sideview of entire whole-mounted cardioids (upper Z-direction) and two single-plane image slices in the middle of each cardioids (bottom two). (a) 200 µm cardioids showed limited cavitation with isolated small cavities. (b) 600 µm cardioids showed large cavity formation resembling early heart chamber. (c) 1000 µm cardioids showed interconnected mesh-like cavitation. Scale bar: 100 µm.

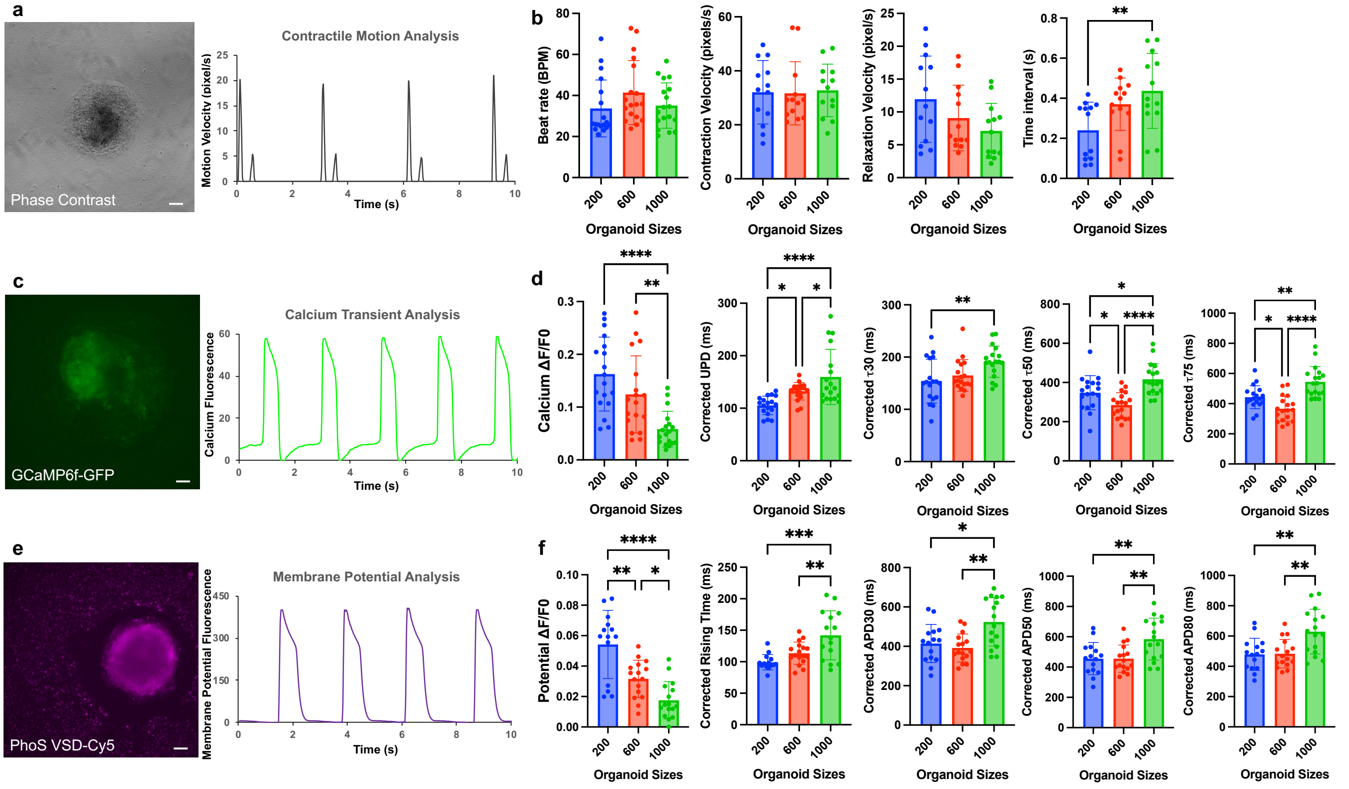

Figure S10. Contractile function analysis of Day 21 micropatterned cardioids with different sizes (related to Figure 5). (a) Representative brightfield image (600 µm cardioids) used for contractile motion analysis, revealing (b) an elongated contraction-relaxation time interval in larger 1000 µm cardioids. (c) Representative GCaMP6f-GFP fluorescence image (600 µm cardioids) for calcium transient analysis. (d) 600 µm cardioids exhibited the shortest calcium decay time, whereas 1000 µm cardioids displayed prolonged calcium transients. (e) Representative PhoS VSD-Cy5 fluorescence image (600 µm cardioids) for membrane potential analysis, indicating that 1000 µm cardioids had an extended action potential duration. Scale bar: 50 µm. Sample size > 10 cardioids from three independent differentiation. Statistical analysis: one-way ANOVA with post-hoc Tukey analysis (*p < 0.05, **p<0.01, ***p<0.001, ****p<0.001).

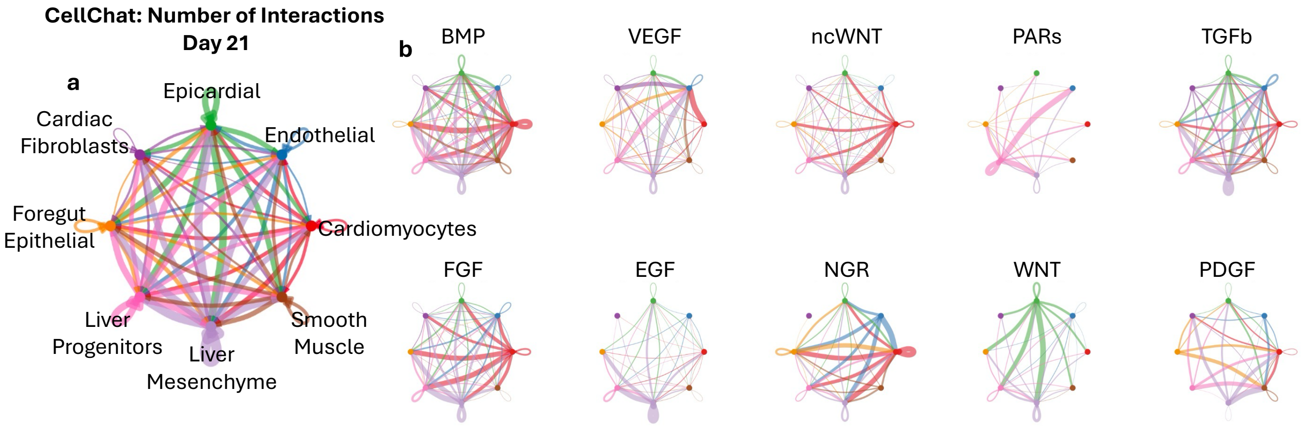

Figure S11. Ligand-receptor analysis on Day 21 for heart-foregut crosstalk (related to Figure 5). (a) Cell-cell interaction networks for all detected signaling pathways. (b) Cell-cell interactions highlighting selected embryogenesis-related signaling pathways.

Supplemental Movies

Movie S1. 3D reconstructed confocal microscopy image of a micropatterned cardioid stained with cardiac troponin (TNNI2) and vimentin (VIM).
Movie S2. Cross-sectioning of 3D reconstructed two-photon microscopy image of a micropatterned cardioid generated from TNNI2-GFP hiPSC reporter line and stained with endodermal marker HNF3A.
Movie S3. Brightfield video of a beating 600 µm cardioid.
Movie S4. Fluorescent video of a beating 600 µm cardioid generated from GCaMP6f-GFP reporter hiPSC line for calcium transient analysis.
Movie S5. Fluorescent video of a beating 600 µm cardioid stained by membrane potential dye (PhoS VSD-Cy5) for action potential analysis.

Supplemental Table

**Table S1: Antibodies used in the study**			
Primary antibody			
CDH5	V1514-200ul	Sigma	1:100
PECAM1	PA5-32321	Invitrogen	1:100
HNF3B	sc-374376	Santa Cruz	1:100
HNF3A	ab170933	Abcam	1:100
TBX18	sc-130428	Santa Cruz	1:100
WT1	PA5-16879	Invitrogen	1:50
TNNI	ab47003	Abcam	1:200
TNNT2	MA5-12960	Invitrogen	1:200
BeRST 1	PhoS1-10	PhotoSwitch Bioscience	1:100
Secondary Antibody			
Goat anti-Mouse IgG, Alexa Fluor 488	A-11029	Invitrogen	1:200
Goat anti-Rabbit IgG, Alexa Fluor 488	A11008	Invitrogen	1:200
Donkey anti-Goat IgG, Alexa Fluor 546	A11056	Invitrogen	1:200
Goat anti-Mouse IgG, Alexa Fluor 546	A11003	Invitrogen	1:200
Goat anti-Rabbit IgG, Alexa Fluor 546	A11010	Invitrogen	1:200
